# Enhancing the in vitro anticancer efficacy of Paclitaxel against triple-negative breast cancer via an exopolysaccharide from a haloalkaliphilic archaeal strain

**DOI:** 10.1038/s41598-025-16243-7

**Published:** 2025-09-23

**Authors:** Afnan Taghian, Mona E.M. Mabrouk, Soraya Sabry, Hanan Ghozlan, Fatma Elsayed

**Affiliations:** 1 Faculty of Science, Botany and Microbiology Department, Alexandria University, Alexandria, Egypt; 2 Faculty of Science, Botany and Microbiology Department, Damanhour University, Damanhur, Egypt; 3 Medical Research Institute, Alexandria University, Alexandria, Egypt

**Keywords:** Haloarchaea, *Natrialba chahannaoensis*, Exopolysaccharides, Poly-β-hydroxyalaknoates, Antioxidant, Anticancer, Microbiology, Drug discovery, Toxicology

## Abstract

Exopolysaccharides (EPSs) produced by haloalkaliphilic archaea exhibit unique properties contributing to varied industrial and medicinal applications. This study explored the anticancer therapeutic potential of an EPS derived from a haloalkaliphilic archaeal strain, which has not been previously isolated from solar salterns on the northwestern Mediterranean Egyptian coast. The selected isolate was identified as *Natrialba chahannaoensis* BG8. A Plackett–Burman (PB) fractional factorial design determined NaCl as the only significant variable positively affecting EPS production. The partially purified EPS analytical characterization revealed a carbohydrate content of 75.16 ± 2.1%, with Fourier transform infrared (FT-IR) spectroscopy; moreover, GC–MS suggested a heteropolysaccharide nature. For the first time, a neutral red uptake (NRU) assay revealed the anticancer effect of EPS against multiple human cancerous cell lines; A-431 epidermoid cancer (IC_50_= 8.8 mg/mL), MCF-7 breast cancer (IC_50_= 12.7 mg/mL), MDA-MB-231 triple-negative breast cancer (TNBC) (IC_50_= 9.4 mg/mL), and HCT-116 colorectal cancer (IC_50_= 10.4 mg/mL) cells. Additionally, EPS exhibited previously reported anti-hepatoblastoma activity against HepG-2 cells (IC_50_= 21.2 mg/mL). The cell cycle analysis results suggested that the antiproliferative effect on MDA-MB-231 cells occurred through S phase arrest. Notably, synergistic interactions between EPS and the anticancer drug paclitaxel (PXL) were recorded in MDA-MB-231 cells via the Chou‒Talalay approach. Furthermore, an unpaired Student’s t test disclosed that EPS induced a significant rise in the apoptosis marker caspase-3 (casp-3). This increase was higher than PXL alone and combined with EPS. However, the combined treatment exceeded PXL in significantly reducing MDA-MB-231 cells’ migratory potentials, as evidenced by wound healing and matrix metalloproteinase-9 (MMP-9) determination assays. It also reduced the levels of the oxidative stress marker malondialdehyde (MDA). The EPS of *N. chahannaoensis* BG8 displayed a pro-apoptotic action against the TNBC cells MDA-MB-231, surpassing that of PXL. Furthermore, PXL–EPS combination reduced PXL-associated toxicity and increased metastasis control. These findings put EPS as a recommended safe complementary therapy, more effective than PXL monotherapy.

## Introduction

The bioactive compounds produced by haloarchaea, in contrast to their counterparts synthesized by other microorganisms, have attracted interest because of the extreme living conditions in haloarchaeal ecosystems, which enhanced their ability to synthesize structurally and functionally distinct metabolites^1^. Haloarchaea require 10% (w/v) salt, with optimal growth at 20–30%. Haloarchaea grow optimally at neutral to alkaline pH values and at 45 °C, with some species exhibiting exceptional growth above 50 °C^2^.

Haloarchaea exhibit diverse metabolic strategies to adapt to harsh conditions, encompassing the synthesis of intracellular granules such as poly-β-hydroxyalkanoates (PHAs) and bioplastic granules and gas vesicles, as well as different metabolites, including enzymes, halocins, and carotenoids. These compounds are of high biotechnological, medicinal, and pharmaceutical significance with wide-ranging bioactivities, including anticancer, antimicrobial, antioxidant, and anti-inflammatory. Moreover, several recent studies reported environmental, medicinal, and pharmaceutical applications of haloarchaeal gas vacuoles, liposomes^3–6^, and nanoparticles synthesized in the course of heavy metal detoxification with various applications, especially as nanodrug carriers^7,8^.

Microbial polymers, including haloarchaeal polymers^1^, have received attention for their renewability, distinct attributes, and availability as sustainable alternatives to traditional petroleum-based polymers. Bacteria and archaebacteria can efficiently utilize various carbon sources for the synthesis of various polymers, including extracellular polysaccharides (EPSs) and intracellular polyesters, polyamides, lipids, polyphosphates, and inorganic polyanhydrides, with varying structural and functional properties^9–11^. Haloarchaea are reported for their ability to synthesize high-quality PHAs with low melting temperatures, crystallinity, polydispersity, and high molecular mass and processability^3,12,13^.

EPSs are structurally heterogeneous polymers composed of homo or hetero carbohydrates together with organic and inorganic substituents^14,15^. Sulfated EPSs receive special concern because of their anticancer action, including their inhibitory effect on human leukaemic T (Jurkat T) cell activity^16^, and consist principally of sugars and proteins^17^.

EPSs produced by extremophiles, including haloarchaea such as *Haloarcula*,* Halorubrum*,* Haloferax*, and *Haloterrigena*, have received substantial attention^18–21^. EPSs from halophilic bacteria and archaea exhibit distinct structural diversity and bioactivities^22,23^ and demonstrate interesting functional properties including gelling, stabilizing, emulsifying, and film-forming attributes^20,24^. Moreover, some halophilic EPSs exhibit health benefits as antioxidant, anticancer, anticytotoxic, prebiotic, and antimicrobial agents^16,25–28^, while their potential antiviral effect on SARS-CoV-2 was highlighted in a recent study^6^.

Despite the intense research on the anticancer activity of halobacteria EPSs, only a few studies have discussed their anticancer attributes^1^. The report by Safarpour et al. (2019)^29^ was the first to address the anticancer action of haloarchaeal supernatant metabolites, where the supernatant metabolites of *Halobacterium salinarum* IBRC M10715 showed in vitro as well as in vivo antiprostate cancer activity. Additionally, Kowalewicz-Kulbat et al. (2023)^30^ reported the anti-ovarian cancer effects of supernatant metabolites of two haloarcaeal strains *Halorhabdus rudnickae* and *Natrinema salaciae.*

In light of the ongoing efforts to investigate microbial EPSs as adjuvant chemotherapy, few reports have described the possibility of applying haloarchaeal EPSs as complementary anticancer therapies. This includes the study by Chouchane et al. (2020)^31^, who demonstrated the anti-hepatoblastoma attribute of haloarchaeal EPS produced by *Halogeometricum borinquense* 52 isolated from an atypical Tunisian environment. Interestingly, the study highlighted the EPS role as an adjuvant, antifatigue anticancer therapy where the EPS antioxidant potential was proven to alleviate oxidative stress; hence, the fatigue associated normally with chemotherapy, hormonal therapy, and radiotherapy treatment in cancer patients.

Extensive research have been made to enhance the anticancer action of paclitaxel (PXL) which is a pro-apoptotic cell cycle-addressing chemotherapeutic agent with a microtubule-stabilizing effect and an inhibitory action on the microtubule reorganization essential for vital interphase and mitotic cellular functions^32^. As one of the most popular anticancer medications, PXL is prescribed to treat a variety of malignancies, including breast cancer, despite the development of resistance to the treatment, which is basically influenced by ATP-binding cassette (ABC) transporters, microRNAs (miRNAs), or gene mutations. PXL side effects, including hypersensitivity or peripheral neuropathy related to the solvent utilized to overcome PXL’s poor solubility, were also reported^33^.

In the same context, PXL-based chemotherapy represents the mainstream treatment for triple-negative breast cancer (TNBC), known to be negative for the receptors of both estrogen and progesterone and human epidermal growth factor receptor-2. In addition, TNBC is E-cadherin negative; expresses mutated *p53*; is considered the most aggressive breast cancer subtype; has the worst early recurrence rate, resistance, metastasis, and mortality; and represents 15–20% of the five breast cancer subtypes^34,35^. Although PXL is the first-line chemotherapy for TNBC treatment, its therapeutic role is limited by its low solubility in addition to its toxicity and hypersensitivity^36^. Studies investigating the possibility of using lower doses of PXL for realizing more effective and safer chemotherapy include the report by Li et al. (2018)^37^, which revealed its downregulating effect on c-Myc and P-c-Myc expression, thereby resulting in arresting the cellular cycle in the G_0_/G_1_ phase in the colorectal carcinoma cell line HCT-116. Furthermore, Gündoğdu (2020) reported^38^, through clonogenic assays, that PXL in combination with olaparib significantly inhibited the survival of colorectal carcinoma patients compared with each of them separately; moreover, western blotting and immunofluorescence showed that olaparib–PXL combination significantly increased the DNA damage levels elicited by higher doses of PXL.

In view of the limited research on bioactive compounds from haloarchaea EPSs, the current work aims to reveal the anticancer attributes of EPS from the haloarchaeal strain *Natrialba chahannaoensis* BG8 isolated from the northwestern coastal solar salterns of Egypt, with a special focus on EPS’s potential role as a safe, effective complementary therapy to PXL for treating TNBC.

## Materials and methods

### Sample collection for enrichment isolation

Water samples (100 mL) were collected in sterile containers from the Mediterranean solar salterns on the northwestern coast of Egypt (latitude 31.153056 N, longitude 29.898611 E) and transported at 4 °C to the laboratory. Horikoshi medium, designed for the isolation of alkaliphilic and alkalitolerant bacteria and/or archaea, was modified by adding NaCl for the isolation of haloalkaliphilic and haloalkalitolerant bacteria and/or archaea. The modified Horikoshi medium was composed in g/L as follows: glucose, 10; K_2_HPO_4_, 1; peptone, 5; MgSO_4_.7H_2_O, 0.2; Na_2_CO_3_, 10; NaCl, 240; and yeast extract, 5. The pH value was set to 9. Agar-agar (20 g/L) was added for plate preparation (Horikoshi, 1999)^39^. Ten milliliters of each sample was inoculated into 100 mL of medium aliquots dispensed in 250 mL Erlenmeyer flasks. Incubation proceeded at 37 °C under light shaken conditions at 150 rpm for 7 days, after which microbial colonies were isolated on modified Horikoshi agar plates using the streak plate method under the same culture conditions. Morphologically distinct colonies were checked daily.

### Culture conditions with optimized salinity for growth

The optimal salinity affecting the growth of isolates was determined using NaCl concentrations varied at 1, 2, 3, 4, and 5 M in modified Horikoshi medium (pH 9). After a 7-day incubation at 37 °C under light, shaking conditions at 150 rpm, the OD_600_ values were recorded. On the basis of this step, the salinity required for the next phase of EPS production screening was determined, and the same culture conditions with the optimized salinity were adopted for preculture preparation, growth, and EPS production.

### EPS production screening and partial purification

The isolates were screened for EPS production in modified Horikoshi broth (4 M NaCl, pH 9). A 2% inoculum of each preculture (OD_600_ ≃ 0.9) was used to inoculate the corresponding EPS production medium aliquots prior to incubation at 37 °C for 7 days under light, shaking conditions at 150 rpm. EPS precipitation from the culture supernatant was achieved by cold ethanol addition (three volumes), followed by incubation at 4 °C for 1 h and centrifugation at 5000 rpm for 15 min. The resulting EPS pellets were dried overnight^40^. Following partial purification, the solubility of the dried partially purified EPS was tested in water, ethyl acetate, chloroform, ethanol, and methanol.

### Phenotypic and genotypic characterization of the selected study isolate

The isolate with the greatest EPS yield was selected and was phenotypically characterized on the basis of its growth conditions, colony macroscopic features, and Gram reaction. The isolate was genotypically characterized using 16S rRNA gene sequence analysis. For this purpose, the cell pellet was washed with 0.9% NaCl solution before DNA extraction using SolGent purification beads (SolGent, South Korea).

Following DNA extraction, the polymerase chain reaction (PCR) amplification of the 16S rRNA gene was performed using universal primers forward: 27 F 5′-AGA GTT TGA TCC TGG CTC AG-3′, and reverse: 1492R 5′-GGT TAC CTT GTT ACG ACT T-3′. The amplified gene was outsourced to SolGent Company (USA). Sequencing was conducted using a BigDye^®^ Terminator v3.1 Cycle Sequencing Kit, the ABI 3730XL DNA Analyser, and a Veriti™ 96-Well Thermal Cycler Sequencer (Applied Biosystems, USA). The 16S rRNA gene sequence analysis was conducted utilizing BLASTn, the basic local alignment algorithm from the National Center for Biotechnology Information (NCBI)^41^, and the similarity percentage with other sequences in the NCBI database was then determined. The sequence was submitted to the NCBI GenBank under the accession number OR738703. The gene sequences of 16S rRNA of *N. chahannaoensis* BG8 and the closest relatives were uploaded onto Clustal W^42^ with default parameters, an algorithm for multiple sequence alignment. A phylogenetic tree describing the phylogenetic relationships between *N. chahannaoensis* BG8 and other archaeal strains was constructed using MEGA X software^43^.

### Screening some factors affecting enhanced EPS production

#### One-factor-at-a-time (OFAT) approach

 The OFAT principle was applied to examine the impacts of various nitrogen and carbon sources on increased EPS yield. Carbon sources, including arabinose, fructose, galactose, lactose, sucrose, and starch, were tested individually at a concentration of 10 g/L in the modified Horikoshi broth in replacement of and in comparison with its native component glucose. Nitrogen sources, which included yeast extract, beef extract, soybean, peptone, and ammonium chloride (NH_4_Cl), were individually added as 1.2 g of nitrogen equivalent to the modified Horikoshi broth in comparison with its native components, peptone and yeast extract. The media were dispensed as 50 mL aliquots in 100 mL Erlenmeyer flasks before being inoculated with 2% (v/v) preculture. Medium and culture conditions were described in Sect. 2.2. Following the 7-day incubation period, for each culture, the OD_600_ was recorded, and the EPS was precipitated, dried, and estimated in g%.

#### Plackett–Burman design

Following the OFAT experiment, more factors were tested for their significant effects on enhanced EPS yield using the lowest number of experimental trials by implementing a Plackett–Burman fractional factorial design (PB) to screen seven selected variables via 12 randomized factorial trials. Modified Horikoshi medium was used as a basal production medium representing the central levels of the investigated factors. Assuming that n is the number of factorial runs, and that PB allows testing an n-1 number of factors with a minimum of n runs, where n should be a multiple of four^44^, a 12-trial matrix (Table 1) was created by Minitab 14 software (Minitab Inc., Pennsylvania, State College, PA, USA) to represent the randomized factorial experiments intended for screening the seven variables—glucose, yeast extract, peptone, NaCl, K_2_HPO_4_, MgSO_4_.7H_2_O, and inoculum size—were each studied at low and high levels, designated as −1 and + 1, as described in Table 2.

The EPS production media used in the factorial experiments were prepared as 50 mL aliquots dispensed in Erlenmeyer flasks (100 mL). After 7 days of incubation, the EPS yield (g%) was recorded as the response of each trial. The main effects were calculated mathematically by subtracting the averaged measurements representing the low levels (−1) from the averaged measurements representing the high levels (+ 1) of each variable^44^. Regression coefficient calculations and additional main effects analysis for estimating the statistical significance of each independent variable on EPS production were conducted using Minitab 14 software, which confirmed the mathematical main effect analysis through running an analysis of variance (ANOVA). On the basis of the main effect analysis, the suggested optimized medium model was verified by comparing the levels of EPS production in the basal, optimized, and anti-optimized media. The optimized medium is normally prepared by including the significant variables at the levels positively affecting the response, whereas the anti-optimized medium is prepared by including the significant variables at the levels negatively affecting the response.

**Table 1:** Plackett–Burman 12-trial matrix for improving EPS production by *N. chahannaoensis* BG8.

Run no.	Mg SO_4_	NaCl	K_2_HPO_4_	Peptone	Yeast extract	Glucose	Inoculum size %
1	1	−1	1	−1	−1	1	−1
2	−1	−1	−1	−1	1	1	1
3	−1	−1	1	1	−1	−1	1
4	−1	1	1	−1	1	1	−1
5	1	−1	−1	1	1	1	1
6	−1	1	1	1	−1	1	1
7	1	1	1	−1	1	−1	1
8	1	−1	1	1	1	−1	−1
9	−1	1	−1	1	1	−1	−1
10	1	1	−1	1	−1	1	−1
11	1	1	−1	−1	−1	−1	1
12	−1	−1	−1	−1	−1	−1	−1

**Table 2:** Plackett–Burman test of variable levels.

Factor	High level (+)	Basal level (0)	Low level (-)
Glucose (g/L)	15	10	5
Inoculum size (%)	3	2	1
K_2_HPO_4_ (g/L)	1.5	1	0.5
Yeast extract (g/L)	7.5	5	2.5
Peptone (g/L)	7.5	5	2.5
NaCl (g/L)	300	240	180
MgSO_4_.7H_2_O (g/L)	0.3	0.2	0.1

### Analytical characterization of the EPS

Samples intended for the determination of carbohydrate, protein, and lipid contents, along with Fourier transform infrared spectroscopy (FT-IR), were sent to the Central Laboratory, Faculty of Science, Alexandria University, whereas samples intended for Gas chromatography–Mass spectrometry (GC–MS) were sent to the Functional and Therapeutic Laboratory, Faculty of Agriculture, Alexandria University.

#### Carbohydrate, lipid, and protein contents

The content of carbohydrate in 100 mg of the EPS was quantified employing the phenol-sulphuric acid protocol defined by Stojilkovski et al. (2018)^45^. Lipids were extracted using a chloroform‒methanol mixture (2:1), and their content was determined gravimetrically as described by Daugherty et al. (1983)^46^. Protein content was assessed via the Bradford assay^47^.

#### Fourier transform infrared spectroscopy (FT-IR)

This analysis was carried out to identify the functional groups in the EPS as described by Arayes et al. (2023)^48^, using a Bruker Tensor 37 FT-IR spectrometer (Germany). Two milligrams of EPS powder were ground in 200 mg of spectroscopy-grade potassium bromide (Sigma–Aldrich, USA) and pressed into a pellet prior to measuring the spectra in transmittance mode within the range of 400–4000 cm^−1^ with 4 cm^−1^ resolution. The analysis was performed using the Bruker Optics software OPUS 3.1.

#### Gas chromatography–mass spectrometry (GC–MS)

To reveal the monosaccharide profile of the EPS of *N. chahannaoensis* BG8, 5 mg of the dried EPS was hydrolysed with 2 mL of trifluoroacetic acid (2 M) for 2 h at 120 °C. Hydrolysate reduction was accomplished using KBH_4_ dissolved in NH_4_OH, which was followed by lyophilization and trimethylsilylation by the addition of 80 µL of N, O-bis(trimethylsilyl)trifluoroacetamide and 20 µL of trimethylchlorosilane. A 1-h incubation proceeded at 65 °C^49–51^.

A 0.5 L sample was injected into the Thermo Scientific GC-TSQ mass spectrometer (USA) with an autosampler with a TG-5MS capillary column having dimensions of 30 m length **×** 0.25 mm inner diameter **×** 0.25 m film thickness. Helium was used as a carrier gas with a flow rate of 1 mL/min. The applied chromatographic conditions included holding an initial column temperature at 40 °C for 1.5 min, increasing the temperature to 130 °C at a rate of 40 °C/min; then, increasing it to 290 °C at 8 °C/min for 5 min. The identification of the separated fractions was based on the highest matching ratio with multiple standard digital mass spectral data libraries, including Wiley Registry 8e, mainlib, and replib^49^.

#### Poly-β-hydroxyalkanoate (PHA) detection test

To confirm the possibility of coproduction of PHA and EPS exopolymers by *N. chahannaoensis* BG8, 0.02% alcoholic Sudan black B staining was implemented as a preliminary screening test for lipophilic analytes. The stain was applied for 30 min to a bacterial film prepared from colonies grown on modified Horikoshi agar plates. The excess stain was rinsed off with absolute ethanol prior to microscopic examination, where PHA-producing cells were expected to show a bluish-dark color, whereas cells negative for PHA production were expected to appear colorless^52,53^.

### Anticancer activity of the EPS

#### In vitro culture conditions and cell lines

The anticancer properties of the EPS were inspected against five human cancerous cell lines, the epidermoid squamous cell carcinoma cell line A-431 (RRID: CVCL_0037, ATCC^®^ CRL-1555), the colorectal carcinoma cell line HCT-116 (RRID: CVCL_0291, ATCC^®^ CCL-247™), the hepatoblastoma cell line HepG-2 (RRID: CVCL_0027, ATCC^®^ Cat. No. HB-8065™), and the breast carcinoma cell lines MCF-7 (RRID: CVCL_0031, ATCC # HTB-22), and MDA-MB-231 (RRID: CVCL_0062, ATCC HTB-26™). The cells were obtained from Nawah Scientific, Egypt. Principally, the guidelines of the culture of animal cells described by the American Type Culture Collection (ATCC)^54^ were followed. The maintenance of the cultures was performed in high-glucose DMEM (Dulbecco’s modified Eagle’s medium), fortified with glucose (4.5 g/L) and L-glutamine (4 mM) (Lonza, UK). To prepare a complete culture medium, fetal bovine serum (FBS) (MSE, France) was added at a concentration of 10% (v/v). The maintenance of human peripheral blood mononuclear cells (PBMCs) was achieved in complete RPMI-1640 (Roswell Park Memorial Institute-1640) medium (Lonza, UK). PBMCs were isolated from 5 mL of heparinized human blood, as described by Bøyum (1968)^55^, using Histopaque^®^−1077 (Sigma–Aldrich, USA) density gradient separation medium. Incubation was carried out at 5 ± 1% CO_2_, 95 ± 5% humidity, and 37 ± 1 °C in a CO_2_ incubator (Nuaire, Germany).

#### Evaluation of the in vitro anticancer effects of the EPS

A neutral red uptake (NRU) assay was adopted for this purpose as detailed by Repetto et al. (2008) [56]. The cell suspension was dispensed into 96-well plates (1.5 **×** 10^4^ cells/well), incubated under the previously described in vitro culture conditions. After 24 h, the adherent cells were incubated for 48 h with serial twofold dilutions of EPS (50‒3.125 mg/mL in complete culture medium). Before applying the treatment serial dilutions, the EPS solution in complete culture medium was membrane-filtered using a 0.22 μm syringe filter (Thermo Fisher, USA). Thereafter, 100 µL of neutral red solution (0.044 µg/mL in DMEM) (Sigma–Aldrich, USA), filtered using Thermo Fisher syringe filters (0.22 μm, USA), was added after removing the spent medium. Following a 2-h incubation, sterile 0.9% (w/v) saline solution was used for washing, whereas 100 µL of 50% (v/v) ethanol acidified with 1% (v/v) glacial acetic acid was used for dye extraction before measuring the UV–Vis absorbance utilizing a Bio-Rad 96-well microplate spectrophotometer (Japan) at 490 nm.

A NRU assay was also performed to assess the effect of the EPS on the viability of PBMCs as normal cells. In this context, Repetto et al. (2008) [56] protocol was modified. PBMCs were aliquoted at 200 µL (2.5 × 10^5^ cells/well) into the wells of a 96-well plate. The EPS was investigated at three concentrations (50, 25, and 12.5 mg/mL) in RPMI-1640 complete culture medium. Untreated wells were considered medium controls. After 48 h of incubation, the content of each well was pipetted into an Eppendorf tube and centrifuged for 10 min at room temperature at 400 × g. Following discarding the supernatants, the neutral red solution was aliquoted at 100 µL into the Eppendorf tubes and incubated at 37 °C for 2 h. The supernatants were discarded before washing the cell pellets with saline solution (0.9%, w/v), and the acidified ethanol was pipetted into the tubes at 100 µL to extract the internalized dye. The content of each tube was pipetted into a 96-well plate before the UV–Vis absorbance measurement at 490 nm utilizing a 96-well microplate spectrophotometer.

The trials were performed in triplicate, and the anticancer activity was expressed as the concentration triggering half-maximal inhibition (IC_50_) determined through regression analysis of dose‒response curves using CompuSyn software (ComboSyn, Inc., New York, USA)^57^.

#### EPS selectivity

To address the selectivity of the EPS from *N. chahannaoensis* BG8, the selectivity index (SI) value was calculated as the ratio of the EPS IC_50_ value recorded for the PBMCs to the EPS IC_50_ value recorded for each cell line, as defined by Nogueira & Estólio do Rosário (2010)^58^.

#### Cell cycle analysis

To study the effect of the EPS from *N. chahannaoensis* BG8 on the cell cycle profile of MDA-MB-231 cells, the DNA content of untreated and EPS-treated cells was estimated utilizing propidium iodide (PI) nucleic acid staining, before the flow cytometric analysis. For this purpose, MDA-MB-231 cells were cultured until 70% confluency was reached in 6-well plates; then, treated with the IC_50_ (9.4 mg/mL) of EPS. Treated and untreated cells were pelleted after 48 h of incubation, washed twice with cold phosphate-buffered saline (PBS) (pH = 7.4; Lonza, UK), fixed in 1 mL of 90% (v/v) methanol for 60 min, washed, resuspended in 200 µL of the cell cycle reagent (PI/RNase mixture), and incubated in the dark at room temperature for 30 min^59^. The cell suspensions were transferred to 5 mL flow tubes before performing the flow cytometry analysis at 555 nm to calculate the percentage of cells in each phase. Trials were duplicated. The unpaired Student’s t test, assuming equal variances at a significance level of α = 0.05, was performed to assess the significance of EPS-induced changes in the cell cycle distribution.

#### Paclitaxel‒EPS interaction

To assess the potential of applying *N. chahannaoensis* BG8 EPS as a complementary therapy to chemotherapy, its interaction with the standard anticancer drug paclitaxel (PXL) was investigated following the Chou–Talalay median effect method^60,61^ by calculating the combination index (CI) value which denotes synergistic interaction if < 1, additive interaction if CI = 1, whereas CI > 1 infers antagonism. The CI and dose reduction index (DRI) values were calculated for selected combined treatment concentrations using CompuSyn software.

For this purpose, after estimation of the EPS IC_50_ as described earlier, the NRU assay was applied to estimate the IC_50_ of PXL against the human TNBC MDA-MB-231 cells. The NRU assay was subsequently applied to measure the viability of MDA-MB-231 cells after exposure to either the EPS (50–3.125 mg/mL), PXL (0.12–0.004 mg/mL), or their combination for 48 h. In this trial, PXL and EPS single treatments were conducted alongside the combined treatment to detect any shifts in IC_50_ values, thereby minimizing errors in CI calculations. The concentrations used for the combined treatment included the IC_50_ of EPS and PXL, as well as the lower fractions of their IC_50_ values. The highest concentration of the combined treatment was subjected to a nonconstant ratio dilution, as detailed in Table 3. Trials were duplicated.

**Table 3:** The non-constant ratio-diluted concentrations of EPS and PXL in the combined treatments.

EPS extract (mg/mL)	Dose PXL (mg/mL)
25	0.060
12.5	0.060
6.25	0.045
3.13	0.030
1.56	0.019
0.78	0.010
0.39	0.006
0.20	0.003
0.10	0.002
0.05	0.001
0.025	0.0005

#### In vitro individual and combined treatment with EPS and PXL

- Scratch wound healing assay.

This assay was conducted according to Martinotti & Ranzato et al. (2019) method^62^, modified using 24-well plates instead of 6-well plates, to assess the in vitro antimigratory potential of the EPS of *N. chahannaoensis* BG8 against the TNBC MDA-MB-231 cells treated with EPS at 5, 3, or 1 mg/mL in serum-free culture medium. Untreated wells represented the control group. In parallel, a combined treatment group was treated with PXL (0.001 mg/mL) and EPS (1 mg/mL), while the PXL-treated group (0.001 mg/mL) was considered the standard drug control.

Trials were duplicated. ImageJ 1.5a software^63^ was used to analyse the wound pictures at zero, 24, and 48 h. A total of 15–20 wound diameter measurements were recorded per group. The results were expressed as the wound healing percentage relative to the untreated control group. Unpaired Student’s t test, (α = 0.05) assuming unequal variances, was applied to run pairwise comparisons to verify the statistical significance of the potential antimigratory effect of EPS and PXL–EPS treatments compared with both the untreated control and the PXL-treated control groups.

- Oxidative stress, apoptosis, and metastasis biomarker determination.

MDA-MB-231 cells were separately treated with EPS (9.4 mg/mL), PXL (0.060 mg/mL), or a combination of PXL (0.030 mg/mL) and EPS (3 mg/mL). The individual and combined treatments were applied at their respective IC_50,_ as determined by the Chou–Talalay median effect method. After 48 h, the cell pellets and culture supernatants were collected. Malondialdehyde (MDA), a cellular oxidative stress marker reflecting the level of lipid peroxidation and cellular damage indirectly^64^, was determined colorimetrically in the cellular pellet using Novus Malondialdehyde Assay Kit (Novus Biological, USA). Caspase-3 (casp-3) specific activity, an apoptotic marker^65^, was determined colorimetrically in the cell pellet (Sigma Caspase-3 Assay Kit- Sigma–Aldrich, USA), whereas the level of matrix metalloproteinase-9 (MMP-9), a metastasis and angiogenesis marker^66^, was assessed in the supernatant via Elabscience^®^ Human MMP-9 enzyme-linked immunosorbent assay (ELISA) Kit. Trials were duplicated. A pairwise comparison using an unpaired Student’s t test (α = 0.05), with unequal variances assumed, was performed to assess the significance of each treatment compared with the untreated control group.

### Declarations

Blood samples used for PBMCs isolation were obtained from a volunteer research group member.

## Results and discussion

### Isolate screening for EPS production

**Fig. 1:**
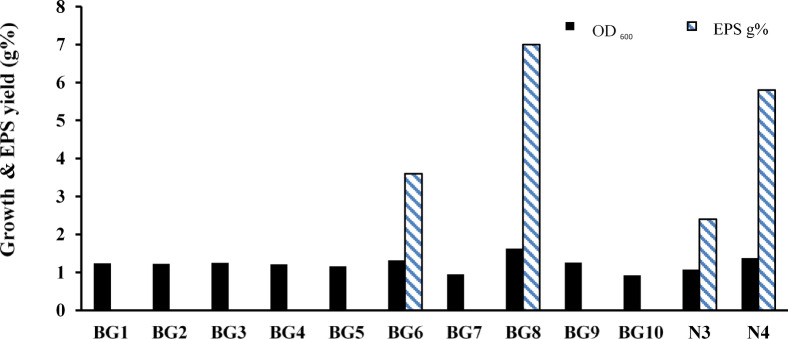
EPS production by the 12 haloalkalitolerant bacterial/archaeal isolates.

The present study aimed to isolate haloalkalitolerant/haloalkaliphilic bacteria or archaea from water samples of solar salterns on the Mediterranean northwest coast of Egypt, using modified Horikoshi medium (pH 9, 4 M NaCl). The isolation step resulted in 12 haloalkalitolerant/haloalkaliphilic isolates, which were screened after 7 days of incubation under the same enrichment isolation conditions for EPS production. The yield of dried EPS, precipitated from each culture supernatant, was recorded in g%, as was the culture OD_600_. BG8 isolate showed the greatest growth (OD_600_ = 1.63) and EPS yield (7 g%) (Fig. 1) and was selected for further experimentation.

### Phenotypic and genotypic characterization of BG8

**Fig. 2:**
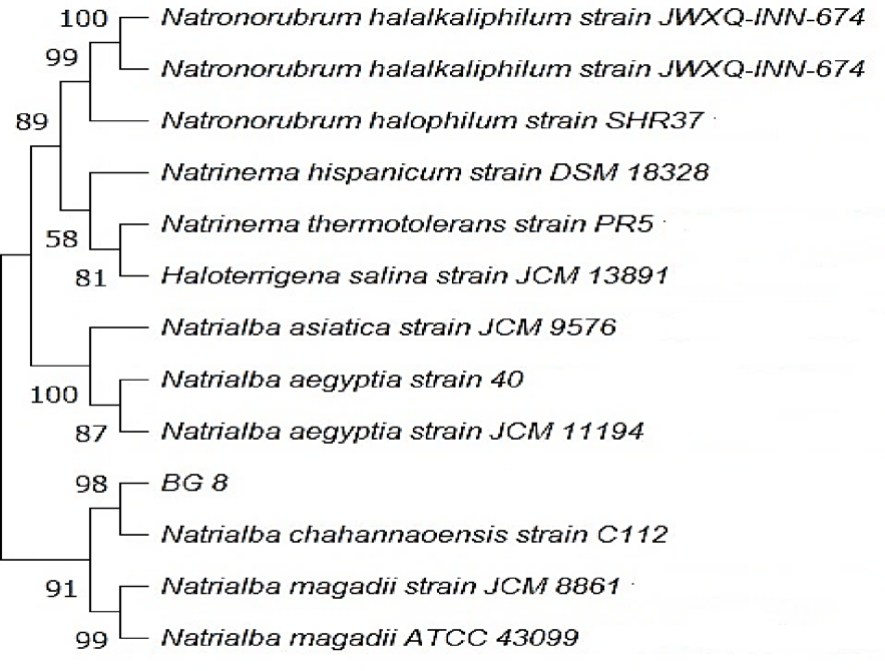
NCBI BLAST-constructed phylogenetic tree of isolate BG8 and its closest neighbors.

BG8 gives small, round, pink colonies. Microscopic examination revealed Gram-negative cocci. It exhibited aerobic growth at 1–4 M NaCl, pH 9, and 4–37 ℃, with optimal growth at 25 ℃ and 4 M NaCl. NaCl and alkaline pH were required for BG8 growth. 16S rRNA gene sequence analysis using NCBI BLASTn alignment revealed sequence similarity to *Natrialba chahannaoensis*; hence, the isolate was named *N. chahannaoensis* BG8, and the sequence was submitted to GenBank with the accession number OR738703. Figure 2 displays the phylogenetic tree describing the phylogenetic relationships among some selected sequences and *N. chahannaoensis* BG8, which clustered with *N. chahannaoensis* C112, confirming the NCBI BLASTn analysis results. To our knowledge, the present report is the first on the isolation of the halorachaeal genus *Natrialba* from Mediterranean solar salterns in Egypt. The haloarchaeon *Natrialba* sp. belongs to the family *Natrialbaceae* within the order *Halobacteriales*^67,68^. In 2001, Xu et al.^69^ reported the novel isolation of *N*. *chahannaoensis* sp. nov., a haloalkaliphilic, aerobic archaeon from soda lakes, Mongolia, China. In parallel, in the same year, Hezayen et al.^70^ reported the isolation of *Natrialba. aegyptiaca* sp. nov., as a novel extreme haloarchaeon from Aswan in Egypt, which produces extracellular polyglutamic acid.

### Screening some factors affecting EPS production

The OFAT approach was applied first with the aim of screening the best carbon and nitrogen sources, separately for EPS production by *N. chahannaoensis* BG8. More variables were tested using the fractional factorial statistical design of Plackett–Burman (PB), which allows screening many variables with the least number of experimental trials in a time-effective way^44^.

#### OFAT model

**Fig. 3:**
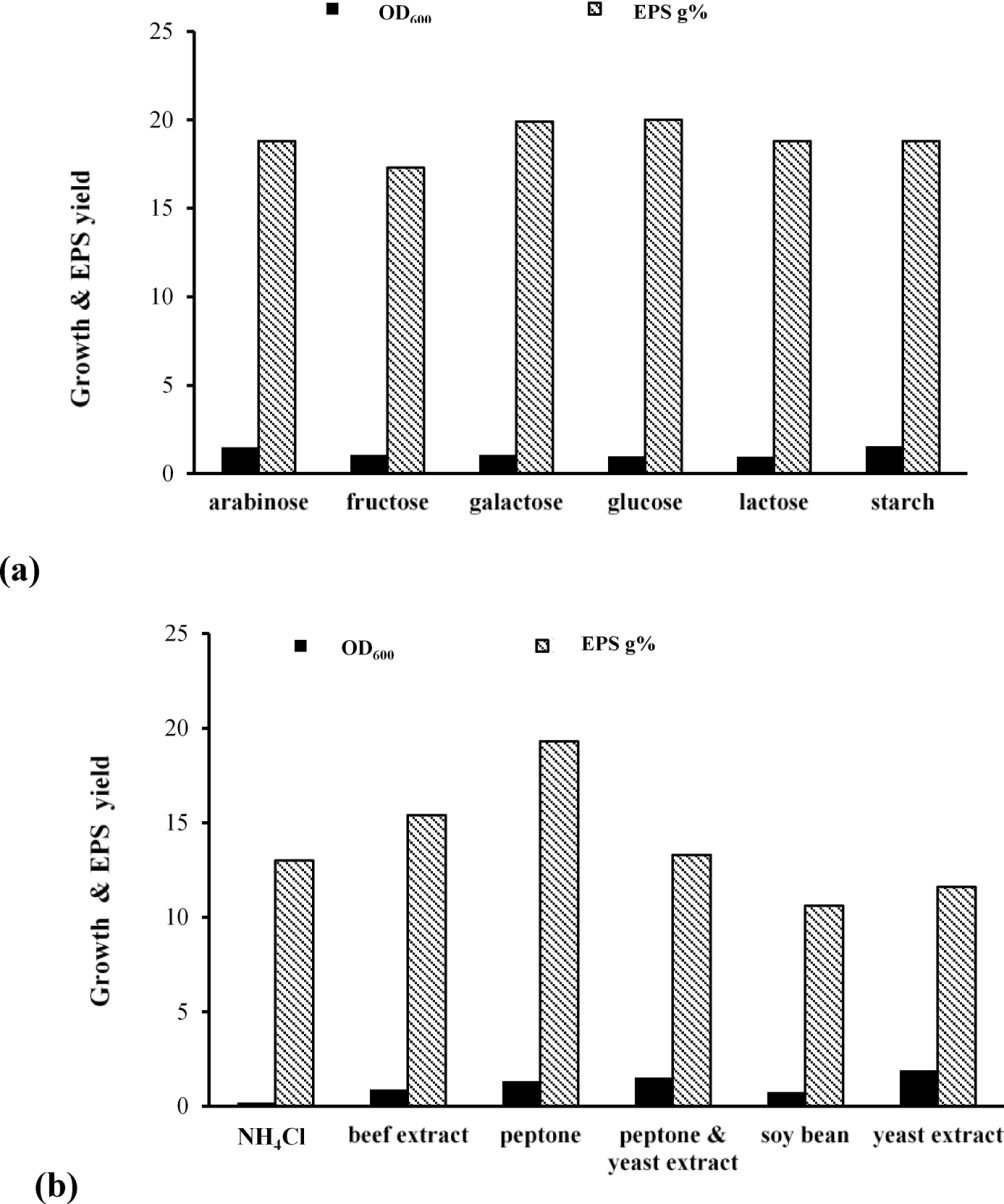
Screening some nutrients for enhanced EPS production by *N. chahannaoensis* BG8 (**a**) carbon and (**b**) nitrogen sources.

In this experiment, galactose, fructose, lactose, starch, and arabinose were separately added at 10 g/L in the modified Horikoshi medium in replacement of and in comparison with the native medium constituent glucose. Figure 3a shows that none of the tested carbon sources demonstrated an advantage over glucose concerning EPS production. Despite the higher OD_600_ values associated with starch and arabinose utilization, no improvement in EPS production was observed. On the other hand, the use of yeast extract triggered the highest growth intensity and the lowest EPS yield, while peptone utilization was associated with the highest EPS yield, which was even greater than the EPS yield resulting from combining yeast extract and peptone in the modified Horikoshi medium (Fig. 3b).

#### Plackett–Burman fractional factorial design (PB)

In this experiment, glucose, peptone, yeast extract, NaCl, K_2_HPO_4_, MgSO_4_.7H_2_O, and inoculum size variables were successfully screened using PB model at two levels, high (+) and low (-), through a 12-run matrix for their significance concerning EPS production as the response variable (Table 4), by *N. chahannaoensis* BG8 after 7 days. Modified Horikoshi medium was used as a basal medium representing the central levels of the tested variables.

**Fig. 4:**
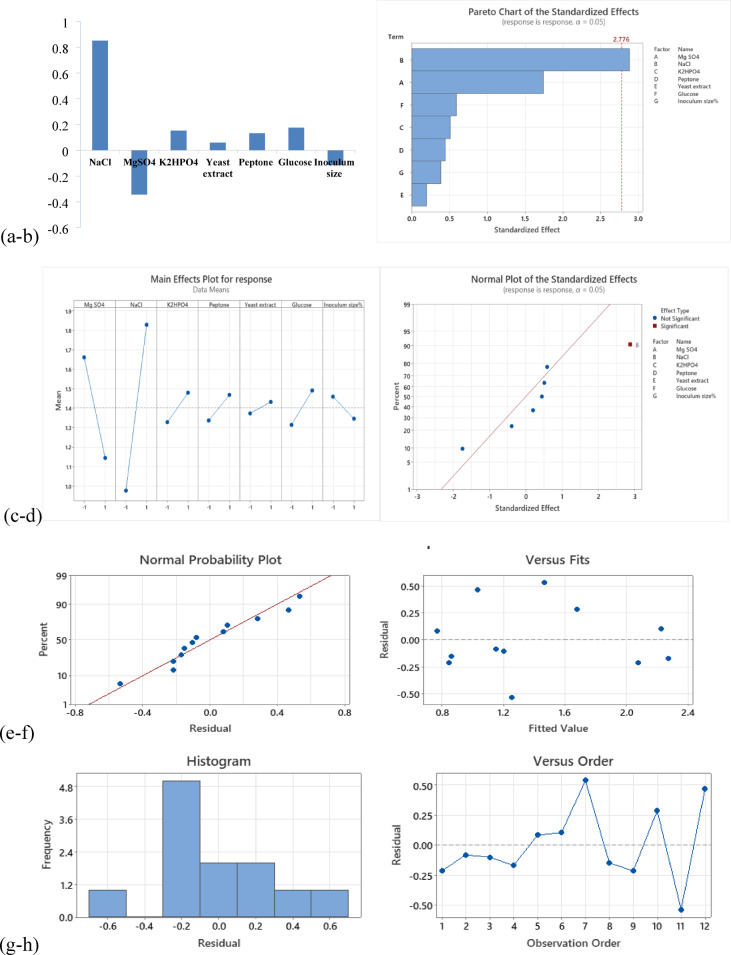
Plackett–Burman analysis of main effects and model residuals. (**a**) Main effect mathematical calculations. (**b**) Pareto chart of main effects statistical significance. (**c**) ANOVA. (**d**) Normal probability plot of effects. (**e**-**h**) Analysis of model response residuals.

On the basis of the mathematical calculation of the main effects, EPS production was most affected by the high level of NaCl, followed by the low MgSO_4_.7H_2_O level (Fig. 4.a), an observation further analysed via Minitab 14 main effect analysis, where the Pareto chart (Fig. 4b) showed that NaCl was the only variable whose effect exceeded the reference statistical significance value at α = 0.05; hence, it was regarded the only significant variable, a result further confirmed by analysis of variance (ANOVA) as shown in the main effect plot, where the high NaCl level appeared to significantly enhance EPS production (*p* value = 0.045) followed by MgSO_4_.7H_2_O which enhanced EPS production at the lower level, unlike the other variables whose representative lines appeared more horizontal; thus, statistically insignificant (Fig. 4c). In agreement with the ANOVA results, the normal plot of effects confirmed that NaCl was the only tested variable which significantly affected EPS production (*p* value = 0.045) at the high level (Fig. 4d).

The model was validated by comparing the mean EPS yield of the 12 factorial experiments (14 g%) with the response of the modified Horikoshi medium cultures as the center point/basal experiments (14.9 g%), which showed close agreement. In addition, the model was further validated through the analysis of the model response residuals which were closely distributed around a straight line as shown in the normal probability plot of residuals (Fig. 4e), were normally distributed in the response residuals histogram plot (Fig. 4g) and randomly scattered around the zero value in the versus plots (Fig. 4f & h). The regression coefficients were estimated by Minitab 14 via ANOVA, and the model regression equation was as follows:

**EPS yield = 1.402 − 0.258 MgSO**_**4**_**0.7 H**_**2**_**O + 0.426 NaCl + 0.076 K**_**2**_**HPO**_**4**_ **+ 0.066 Peptone **

**+ 0.029 Yeast extract + 0.088 Glucose − 0.057 Inoculum size%**.

Interestingly, according to the model regression statistics, the model explained almost 73% of the variation in the EPS yield, as inferred from the R^2^ value, suggesting that PB model fits the data well (Table 5). The ANOVA results of the PB model effects are summarized in Table 6.

**Fig. 5:**
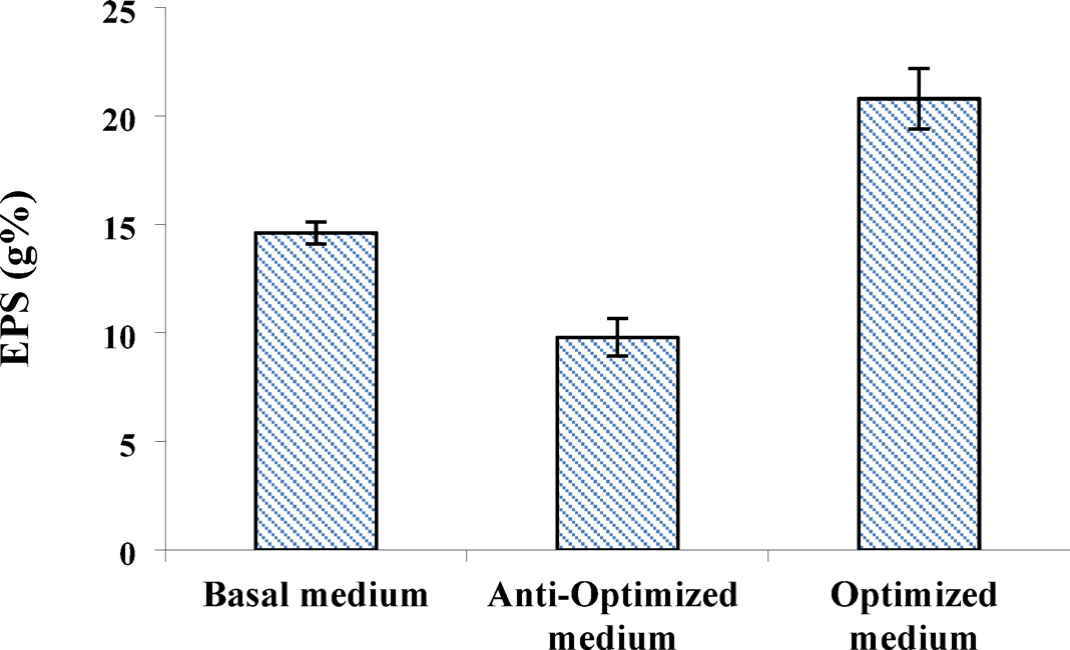
Experimental verification of Plackett–Burman model optimization of EPS production by *N. chahannaoensis* BG8. The error bars refer to the averaged triplicated measurements.

In line with the main effect analysis results, the composition and variable levels of the optimized and anti-optimized conditions were determined (Table 7) for the subsequent step of model experimental verification which disclosed that the EPS yield in the optimized run (20.8 g%) was greater than that in the basal run (14.6 g%), whereas the anti-optimized run (9.8 g%) showed the lowest EPS yield (Fig. 5). PB-optimized conditions were applied for EPS production by *N. chahannaoensis* BG8 for the subsequent experiments.

**Table 4:** Plackett–Burman model runs and responses.

Run no.	MgSO_4_.7H_2_O	NaCl	K_2_HPO_4_	Peptone	Yeast extract	Glucose	Inoculum size %	EPS yield (g%)
1	1	−1	1	−1	−1	1	−1	**0.63**
2	−1	−1	−1	−1	1	1	1	**10.7**
3	−1	−1	1	1	−1	−1	1	**11**
4	−1	1	1	−1	1	1	−1	**21**
5	1	−1	−1	1	1	1	1	**8.5**
6	−1	1	1	1	−1	1	1	**23.3**
7	1	1	1	−1	1	−1	1	**20**
8	1	−1	1	1	1	−1	−1	**7.1**
9	−1	1	−1	1	1	−1	−1	**18.6**
10	1	1	−1	1	−1	1	−1	**19.6**
11	1	1	−1	−1	−1	−1	1	**7.2**
12	−1	−1	−1	−1	−1	−1	−1	**15**
Basal run	0	0	0	0	0	0	0	**14.9**

**Table 5:** Plackett–Burman statistical analysis coded coefficients and regression statistics.

Term	Effect	Coefficient	SE Coefficient	t Value	*p* Value	VIF
Constant		1.402	0.148	9.49	0.001	
MgSO_4_.7H_2_O	−0.515	−0.258	0.148	−1.74	0.156	1.00
NaCl	0.852	0.426	0.148	2.88	0.045	1.00
K_2_HPO_4_	0.152	0.076	0.148	0.51	0.635	1.00
Peptone	0.132	0.066	0.148	0.45	0.679	1.00
Yeast extract	0.058	0.029	0.148	0.20	0.853	1.00
Glucose	0.175	0.088	0.148	0.59	0.586	1.00
Inoculum size %	−0.115	−0.057	0.148	−0.39	0.717	1.00
**Regression statistics**						
	**SE**	**R** ^**2**^				
	0.51	75.52%				

Adj: adjusted, SE: standard error, VIF: variance inflated factor.

**Table 6:** ANOVA of effects.

Source	DF	Adj SS	Adj MS	F Value	*P* Value
Model	7	3.23	0.46	1.76	0.305
Linear	7	3.23	0.46	1.76	0.305
Mg SO_4_	1	0.79	0.79	3.04	0.156
NaCl	1	2.17	2.17	8.30	0.045
K_2_HPO_4_	1	0.07	0.07	0.26	0.635
Peptone	1	0.05	0.05	0.20	0.679
Yeast extract	1	0.01	0.01	0.04	0.853
Glucose	1	0.09	0.09	0.35	0.586
Inoculum size %	1	0.04	0.04	0.15	0.717
Error	4	1.05	0.26		
Total	11	4.28			

DF, degree of freedom; Adj SS, adjusted sum of squares; Adj MS, adjusted mean squares.

**Table 7:** Low (-) and high (+) variable levels in Plackett–Burman optimized and anti-optimized runs.

	Optimized medium	Anti-optimized medium
NaCl (g/L)	+ (300)	− (180)
MgSO_4_.7H_2_O (g/L)	− (0.1)	+ (0.3)
Glucose (g/L)	+ (15)	− (5)
Peptone (g/L)	+ (7.5)	− (2.5)
Yeast extract (g/L)	+ (7.5)	− (2.5)
Inoculum %	− (1)	+ (3)
K_2_HPO_4_ (g/L)	+ (1.5)	− (0.5)

### Analytical characterization of the EPS

EPS produced by *N. chahannaoensis* BG8 was analyzed for carbohydrate, lipid, and protein contents. FT-IR analysis was employed to verify the presence of functional groups, while GC–MS analysis was used to examine the monosaccharide profile of the EPS and sulfated monomers presence.

#### Carbohydrate, lipid, and protein contents

**Fig. 6:**
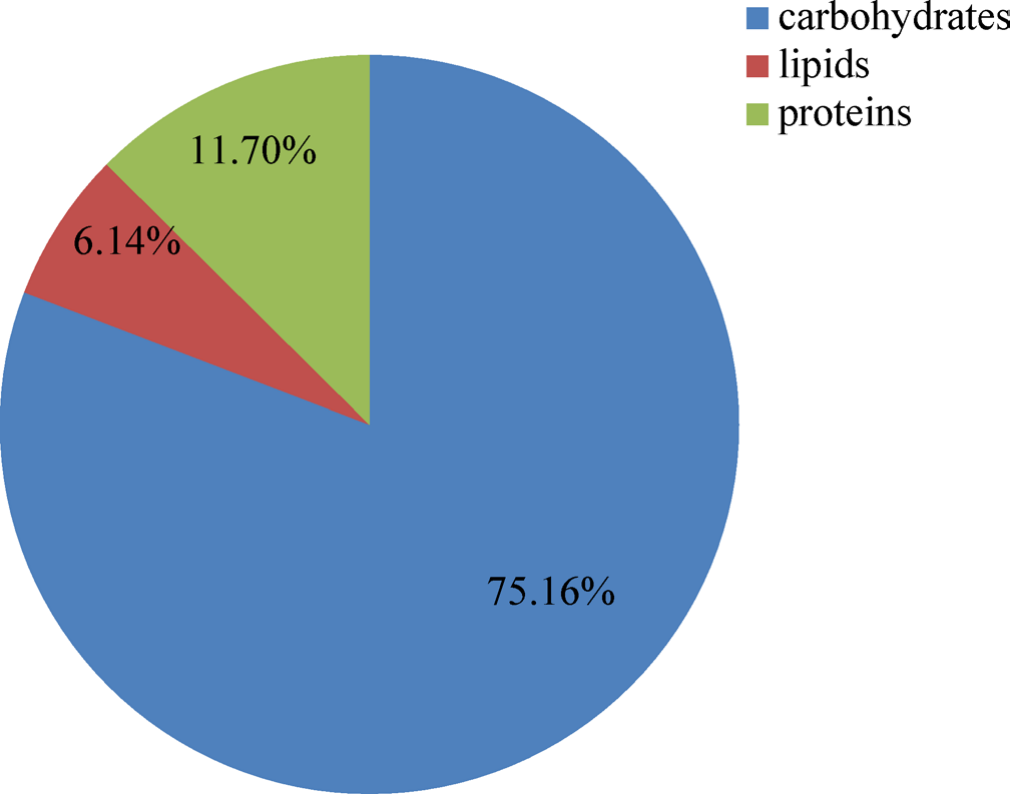
Total carbohydrate, protein, and lipid contents of the EPS of *N. chahannaoensis* BG8.

Carbohydrates accounted for 75.16 ± 2.1 mg% of EPS, whereas the protein content was 11.70 ± 0.32 mg% and lipid content was 6.14 ± 0.3 mg% (Fig. 6).

#### FT-IR

**Fig. 7:**
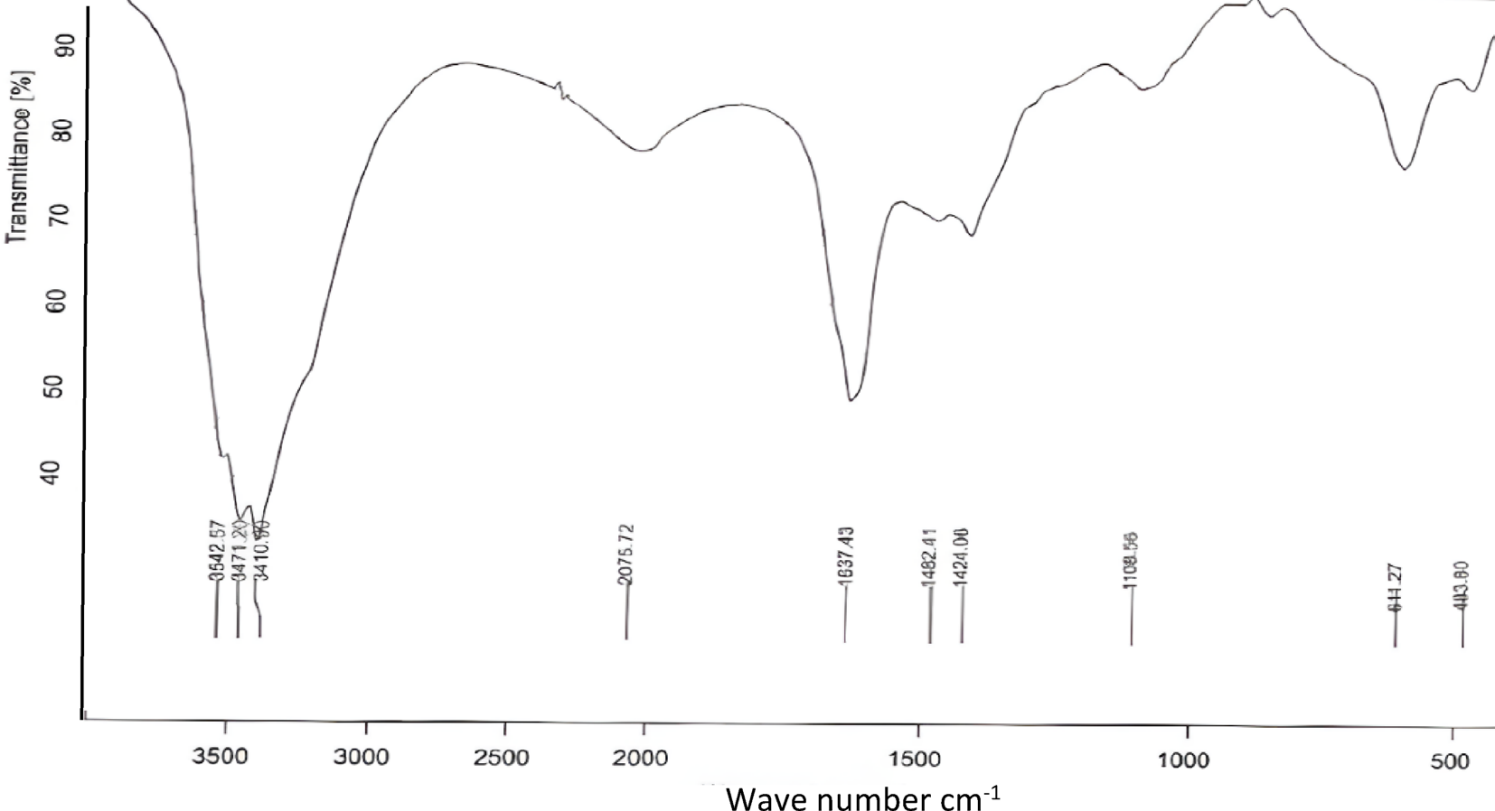
FT-IR analysis of EPS of *N. chahannaoensis* BG8.

FT-IR was performed to define the functional groups and chemical bonds of the EPS of *N. chahannaoensis* BG8. The peaks at 3542, 3471, and 3410 cm^−1^ correspond to the stretching of the hydroxyl group (-OH) characteristic of polysaccharides^71–73^, which may be related to sugar moieties, alcohols, phenols, or amine groups (Fig. 7).

The stretching at 2075 cm^−1^ did not seem common for carbohydrate, protein, or lipid compounds; however, according to Smith (2017)^74^, the alkyne group (C ≡ C) exhibits stretching vibrations in the range 2100–2260 cm^−1^; hence, the 2075 cm^−1^ stretching may represent a substituted alkyne. At 1637 cm^−1^, a sharp peak representing an alkene group (C꞊C)^75^ or amides^76^ was detected. In addition, as stated by Freitas et al. (2009)^77^, the bands at 1593–1662 cm^−1^correspond to the ring stretching vibrations of mannose and galactose. The peak at 1424 cm^−1^ was assigned to (C-H_2_) symmetric bending^78^ as was the O = H bend of esters possibly assigned to C = O and C-O of the COO^79^, whereas the peak at 1482 cm^−1^ was attributed to the (C = C-C) aromatic ring stretch^80^ or saturated lipids^81^ in agreement with the earlier recorded lipid content of 6.14%. The stretching at 1108 cm^−1^ was assigned to the glycosidic linkage group (C-O-C) characteristic of monosaccharides^71,72,82^ or it may be associated with a pyranose configuration^82^ moreover, stretching in this region corresponds to the ether carbonyl group (C-O) and large rings^83^. The peak at 611 cm^−1^ was assigned to alkane group C-H stretching in aliphatic structures^84^ and C-S or S-O groups^85^; nevertheless, data on polysaccharide structures in the region below 800 cm^−1^ are limited and rarely discussed^82^.

To summarize, the FT-IR analysis the presence of hydroxyl, carboxylic, glycosidic, and aliphatic functional groups in the EPS, which is consistent with the structural features of polysaccharides. Similar functional groups have been reported in EPS from *Halomonas smyrnensis* and other haloalkaliphilic species, emphasizing their carbohydrate-dominant composition^48^.

#### GC–MS

GC–MS analysis of the silylation-derivatized EPS components revealed the presence of sugar moieties, including glucose (23.57 min), glucofuranoside (22.66 and 21.83 min), d-xylofuranose (22.78 and 25.46 min), mannopyranoside (16.87, 21.97, 22.26, and 23.85 min), galactopyranoside (17.35, 17.7, and 22.15 min), as well as acetyl glucopyranosyl amine (16.22 min). The observed saccharides peaks, together with the recorded high carbohydrate content of 76% highlight the heteropolysaccharide nature of the EPS; however, on the basis of the peak area % analysis, the sugar moieties showed a relative abundance of < 40% which may be related to transformations occurring as a result of acid hydrolysis and reduction reactions during EPS derivatization^86^. Coincidentally, a peak for ribonic acid lactone was detected (18.41 min), which may have resulted from the transformation of ribose^87^. Arabinopyranose, the cyclic form of arabinose, was also suggested (22.78 min). According to Pan et al. (2010)^88^, EPS with a prominent content of arabinose and ribose is expected to show stronger antioxidant attributes.

Despite the earlier recorded lipid content of 6.14%, the GC–MS analysis showed a more pronounced abundance of lipid moieties, such as oleic acid ester (26.22%, 28.91 min) and palmitic acid ester (19.04%, 25.94 min), which may be attributed to a greater lipid tolerance to the conditions of the adopted GC‒MS derivatization protocol compared with sugars. GC–MS also analysis suggested fatty acid esters with aliphatic saturated side chains (undecynoic, 15.63 min; octadecadiynoic, 7.92 min), whose detection may imply the presence of PHA monomer derivatives^89^ in addition to EPS.

**Fig. 8:**
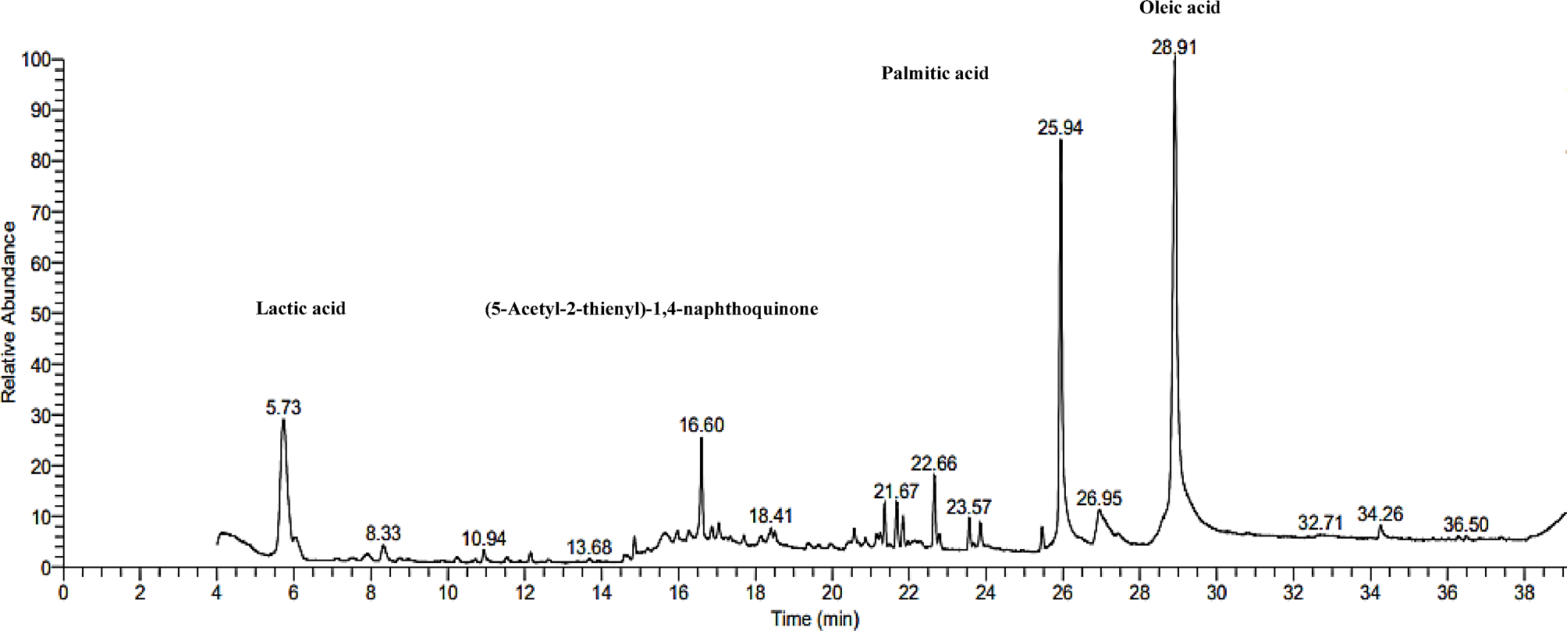
GC–MS full spectrum chromatogram of the EPS of *N. chahannaoensis* BG8.

Following the lipid moiety abundance, the lactic acid peak area was 10.01% at 5.72 min, and a sulfur-containing derivative of acetyl thienyl naphthoquinone showed a 5.11% peak area at 16.60 min. Interestingly, sulfated moieties in EPS are known to be responsible for several bioactivities, including anticoagulant, anti-angiogenic, antiproliferative, and antiviral activities^90^. Moreover, the prominent lactic acid residues peak area of 10.01% may infer the presence of a PLA polymer known for its pharmaceutical applications; however, data on PLA production by haloarchaea are limited in the literature. The full spectrum GC–MS chromatogram is demonstrated in Fig. 8.

#### Poly-β-hydroxyalkanoate (PHA) detection

**Fig. 9:**
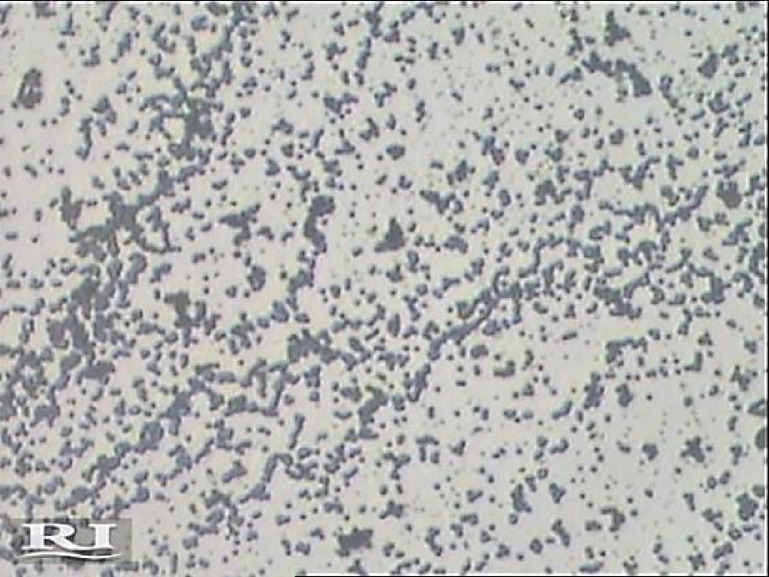
Detection of PHA production by *N*. *chahannaoensis* BG8 using the Sudan black B test. (10x ocular lens x100x objective lens).

The detection of fatty acid esters with aliphatic side chains via GC–MS analysis suggested the production of PHA by *N. chahannaoensis* BG8 in addition to EPS. Accordingly, PHA production was further tested via the Sudan black B staining of a fixed suspension of *N. chahannaoensis* BG8 cells cultured on modified Horikoshi medium plates. Stained cells showed a bluish-dark color upon microscopic inspection as an indicator of the presence of lipophilic metabolites (Fig. 9), which is in agreement with the recorded 6.14% lipid content, the ester groups and lipid moieties suggested by the FT-IR analysis, and the considerably abundant oleic and palmitic fatty acid esters suggested by the GC–MS analysis. Haloarchaea are known to produce high-quality PHA even from cheap organic wastes^91^.

#### EPS analytical structure conclusion

In conclusion, EPS produced by haloalkaliphilic archaeon *N. chahannaoensis* BG8 seems to contain heteropolysaccharide, as determined by FT-IR and GC–MS analysis, suggesting the presence of sugar moieties of glucose, glucofuranoside, d-xylofuranose, mannopyranoside, galactopyranoside, arabinopyranose, and acetyl glucopyranosyl amine.

### Anticancer activity of the EPS

EPS anticancer activity was assessed in aqueous solutions as EPS exhibited greater solubility in water than in ethyl acetate, chloroform, ethanol, or methanol. Interestingly, the GC–MS analysis suggested the presence of a sulfur derivative. Sulfated moieties are known to contribute to anti-angiogenic and antiproliferative activities of EPS^90^; accordingly, the EPS was tested specifically for its potential anticancer attributes.

#### In vitro cytotoxic and/or antiproliferative effects

**Fig. 10:**
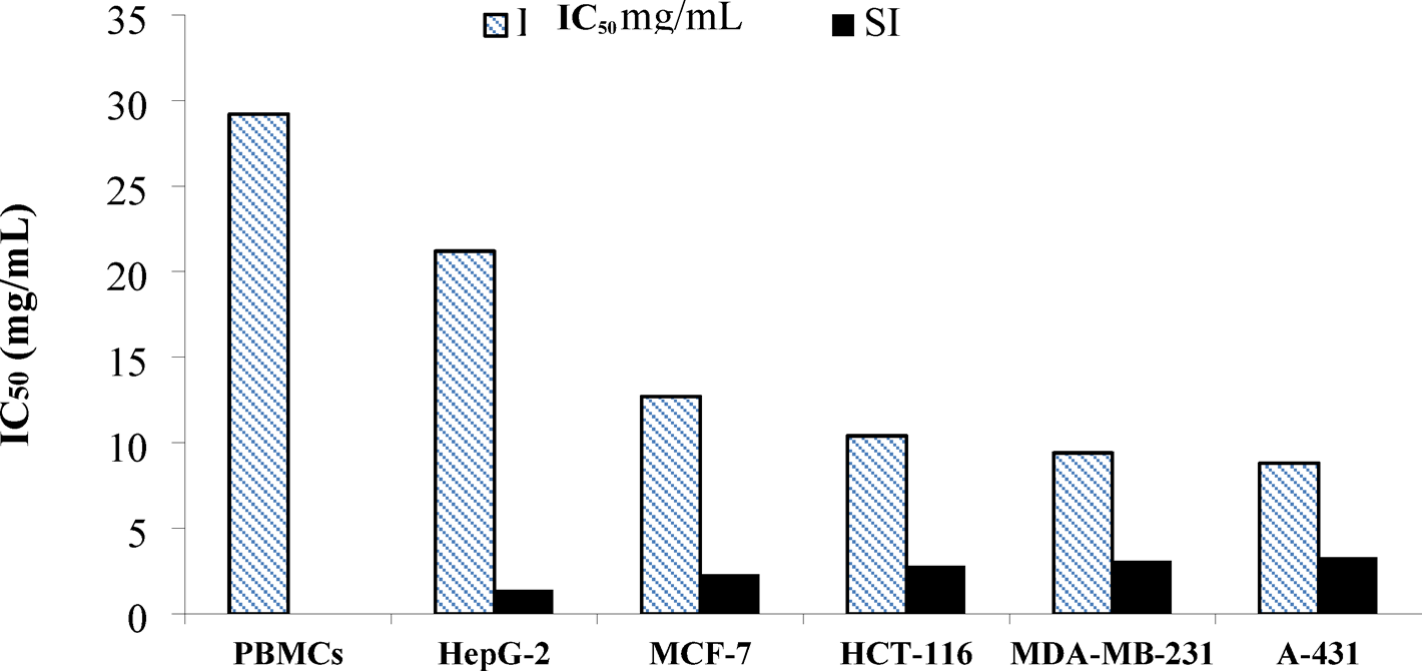
IC_50_ and SI values describing the anticancer activity of the EPS of *N. chahannaoensis* BG8.

The anticancer activity of the EPS of *N. chahannaoensis* BG8 was revealed using the NRU assay after 48 h. Dose‒response curves and the median effect plots illustrating the effects of serial dilutions of the EPS on the viability of the investigated cell lines were constructed via CompuSyn software which calculated IC_50_ values of 8.8, 12.7, 9.4, 10.4, and 21.2 mg/mL of the EPS against the human cell lines of epidermoid squamous carcinoma (A-431), luminal A subtype breast carcinoma (MCF-7), triple-negative breast carcinoma (TNBC) (MDA-MB-231), colorectal carcinoma (HCT-116), and hepatoblastoma (HepG-2) (Fig. 10).

Notably, the EPS exhibited the highest IC_50_ value of 29.2 mg/mL against the normal PBMC model, an observation promising a potentially selective, safe anticancer application of the EPS, a possibility approached by calculating the SI value of EPS corresponding to each cell line, which was calculated as 3.3, 3.1, 2.3, 2.8, and 1.4 against A-431, MDA-MB-231, MCF-7, HCT-116, and HepG-2, respectively; hence, a selective, safe anticancer action of the EPS of *N. chahannaoensis* BG8 towards A-431 and MDA-MB-231 cells followed by MCF-7 and HCT-116 cells was demonstrated. A less selective anticancer action was recorded against HepG-2, as inferred from the SI value of 1.4 below two, not satisfactory as per Nogueira & Estólio do Rosário (2010)^58^ who recommended an acceptable SI value above two for safe application of a natural bioactive compound, whereas Famuyide et al. (2019)^92^ considered an SI value above one satisfactory.

Unlike bacterial EPSs, widely reported in the literature for their anticancer activity^93^, haloarchaeal EPS anticancer activity is either unproven or not sufficiently reported. In this context, the study by Hamidi et al. (2019)^21^ exhibited that the EPS from the haloarchaeaon *Halorubrum* sp. TBZ112 had no anticancer effect at 100–1000 µg/mL on gastric cancer cells (MKN-45) or normal human dermal fibroblasts (HDFs) after 48 h, which was expected considering sulfate groups absence from the investigated EPS. On the other hand, and in agreement with the anti-hepatoblastoma action recorded in the current study for the EPS (IC_50_ = 21.2 mg/mL) of the haloalkaliphilic archaeon *N. chahannaoensis* BG8, Chouchane et al. (2020)^31^ reported an anti-hepatoblastoma effect for the EPS from the haloarchaeal strain *Halogeometricum borinquense* 52, where the maximal inhibition was almost 59% after 72 h of incubation at 600 µg/mL.

To our knowledge, the antibreast cancer action of haloarchaeal EPS has not been reported; however, haloarchaeal metabolites—not clearly defined as EPSs —with antibreast cancer attributes have been observed in the report by Safarpour et al. (2019)^29^, which disclosed that *Halovenus aranensis* and *Halorientalis persicus* supernatant metabolites evoked a significant decrease in the cellular viability of human breast cancer MCF-7 cells at 0.8 µg/mL, whereas *Halopenitus malekzadehii* supernatant metabolites elicited a significant decrease in the cellular viability of human breast cancer cells MDA-MB-468 at 0.8 µg/mL; however, supernatant metabolites synthesized by *Halobacterium salinarum* IBRC M10715 showed the greatest cytotoxic activity (IC_50_ = 0.5 mg/mL) against DU-145 and PC-3 prostate cancer cells.

On the contrary, halobacterial EPSs were clearly reported for antibreast cancer activity as shown in the study by Queiroz et al. (2017)^94^, where the levan polysaccharide from *Halomonas smyrnensis* AAD6T demonstrated an antiproliferative activity through inducing both apoptosis and oxidative stress in human breast cancer cells MCF-7 at 100 µg/mL after 48 h. Also, levan derivatives showed activity against lung, liver, gastric, and breast cancer cells of A-549, HepG-2/C3A, AGS, and MCF-7, respectively. Similarly, an anti-human T leukemia activity^16^ was recorded for halobacterial EPS. Similar to the antibreast cancer, the anticolorectal cancer and anti-epidermoid cancer activities of haloarchaeal EPS are first reported through this study.

#### Cell cycle analysis of MDA-MB-231 cells

**Fig. 11:**
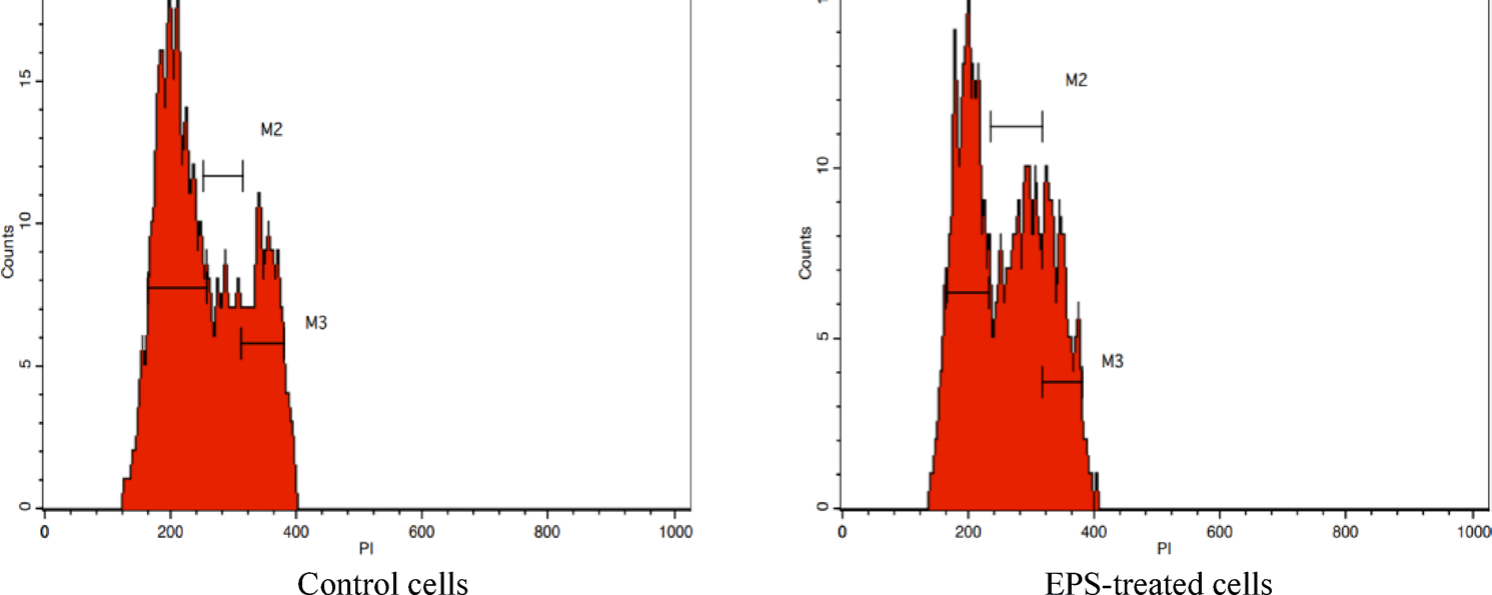
The flow cytometry DNA content histogram describing the EPS influence on MDA-MB-231 cell cycle.

Flow cytometry was used to identify the percentage of cells in each cell cycle phase after 48 h exposure to the IC_50_ (9.4 mg/mL) of the EPS. In the control samples, MDA-MB-231 cells demonstrated the regular cell cycle profile for G0/G1, S, and G2/M phases, where G0/G1 cells represented a majority of 52%, followed by S cells (21%) and G2/M cells (24%). Based on the unpaired Student’s t test (α = 0.05), the EPS insignificantly decreased the percentage of G2 phase cells to 20% while significantly reduced the fraction of MDA-M-231 cells in the G0/G1 phase to 38%, with a corresponding significant increase in the fraction of S phase cells (36%) (Fig. 11), which may be interpreted as an S phase cell cycle arrest which delayed transition into the G2/M then G1 phase of the cycle. Cell cycle arrest at any phase is possibly dictated by DNA damage/mutation and likely associated with apoptosis depending on the level of DNA damage^95^. Furthermore, the reduced number of G0/G1 phase cells has been reported for some anticancer drugs showing a cytostatic antiproliferative action through G1 progression delay and mitosis-interference^96–98^. In brief, a cytostatic antiproliferative effect is concluded.

#### PXL‒EPS interaction against MDA-MB-231 cells

The current study investigated the possibility of applying the EPS of *N. chahannaoensis* BG8 as a complementary therapy to standard anticancer drugs. In light of this, PXL was selected, as an anticancer drug authorized by the Food and Drug Administration as breast cancer chemotherapy^99^, for examining the inhibitory action of its combined treatment with the investigated EPS on MDA-MB-231 cell line representing human TNBC correlated with the highest of breast cancer recurrence risk and death rate during the first five years after diagnosis^34^.

**Fig. 12:**
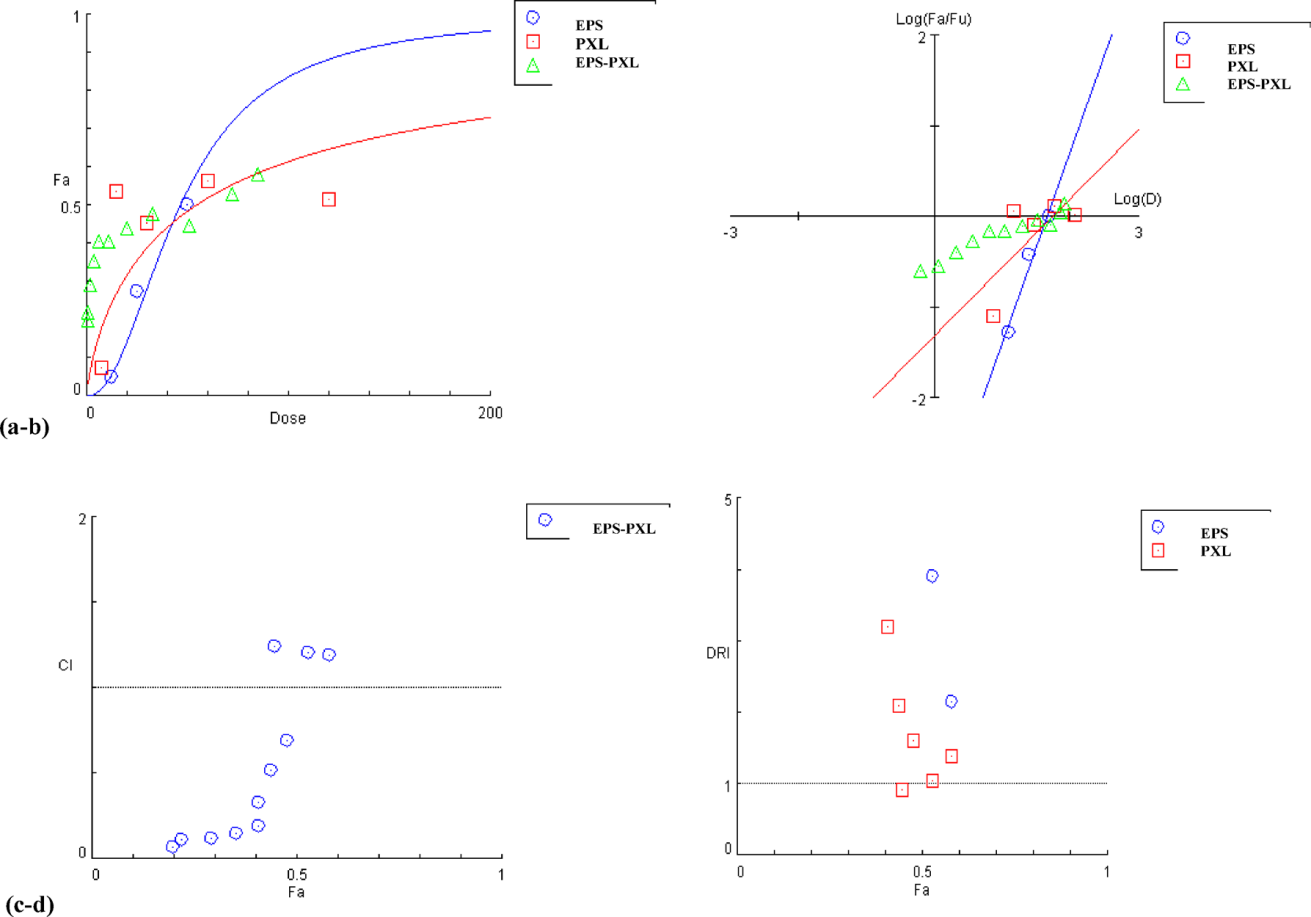
Analysis plots generated via CompuSyn software for PXL‒EPS interactions. (**a**) Dose**‒**effect plot. (**b**) Median-effect plot. (**c**) Combination index plot. (**d**) DRI plot for the combined treatment group. D, dose; Fa, affected inhibition fraction; Fu, unaffected fraction.

Contextually, the NRU assay was conducted to evaluate the viability of MDA-MB-231 cells in response to 48 h exposure to individual and nonconstant ratio combined treatments with EPS (50‒3.125 mg/mL) and PXL (0.12‒0.004 mg/mL). The PXL‒EPS interaction was studied according to the Chou–Talalay approach^60,61^. The median effect plot and the dose‒effect curve, constructed via CompuSyn software, are presented in Fig. 12a & b, whereas Fig. 12c & d represent the CI and DRI plots for nonconstant ratio PXL‒EPS combined treatments. The cellular viability reduction ratio, CI, and DRI values calculated by CompuSyn software are listed in Tables 8 and 9, respectively.

**Table 8:** Combination indices and the inhibitory effects data of PXL‒EPS combined treatments.

EPS (mg/mL)	PXL (mg/mL)	Inhibitory Effect ratio	CI
25	0.060	0.58	1.19
12.5	0.060	0.53	1.21
6.25	0.045	0.45	1.24
3.13	0.030	0.48	0.69
1.56	0.019	0.44	0.52
0.78	0.010	0.41	0.33
0.39	0.006	0.41	0.19
0.2	0.003	0.35	0.15
0.1	0.002	0.29	0.12
0.05	0.001	0.22	0.11
0.024	0.0005	0.20	0.07

In principle, designing combined therapy aims to achieve a synergistic therapeutic action of the standard anticancer drug (chemotherapy) and the suggested complementary therapy; hence, reducing the toxicity and undesired side effects of chemotherapy, and minimizing the possibility of developing resistance in cancer cells (Rosati et al., 2023)^100^.

In this regard, and as concluded from CI values higher than one, the interaction between ESP and PXL was antagonistic at high concentrations of PXL (0.045‒0.060 mg/mL) and EPS (6.25‒25 mg/mL); however, the interaction was synergistic at lower concentrations with CI values ranging from 0.7 to 0.07. Given that synergistic interactions are more relevant therapeutically at concentrations associated with relatively high inhibitory effects^61^, and given the calculated CI values, the EPS and PXL were combined at 3 mg/mL and 0.03 mg/mL, respectively, such concentrations correspond to approximately 50% viability reduction, a CI of 0.69, and a DRI of 14.2 for EPS and 1.6 for PXL, where a DRI value > one indicates a significant dose reduction^61^. Notably, such synergistic interactions come in agreement with the findings by Luque et al. (2024)^101^, who highlighted the potential of haloarchaeal bioactive compounds or extracts to boost the therapeutic efficacy of approved cancer drugs.

**Table 9:** Dose reduction indices (DRIs) of PXL‒EPS combined treatments.

Inhibitory Effect %	EPS (mg/mL)	PXL (mg/mL)	EPS DRI	PXL DRI
58	53.89	0.083	2.16	1.38
53	48.84	0.063	3.91	1.05
45	41.96	0.041	6.71	0.92
48	44.33	0.048	14.19	1.60
44	41.19	0.039	26.37	2.09
41	38.72	0.033	49.56	3.19
41	38.72	0.033	99.13	5.41
35	34.71	0.024	177.71	6.92
29	30.41	0.017	311.38	8.46
22	25.50	0.010	522.29	9.28
19	24.045	0.009	984.88	14.25

#### In vitro treatment with EPS and PXL, individually and in combination

Considering the observed synergistic interaction between EPS and PXL against the TNBC MDA-MB-231 cells, this study aimed to reveal the effect of PXL‒EPS combined treatment on TNBC migratory potential using a scratch wound healing assay; moreover, casp-3 and MMP-9 levels were assessed as biomarkers of apoptosis^65^ and metastasis^66^, respectively. Additionally, the levels of MDA, an oxidative stress marker^64^, were measured.

- **Scratch wound healing assay**.

This assay was conducted to assess the in vitro antimigratory potentials of EPS and PXL–EPS treatments against TNBC MDA-MB-231 cells. Results were expressed as the wound healing percentage relative to the control group.

**Fig. 13:**
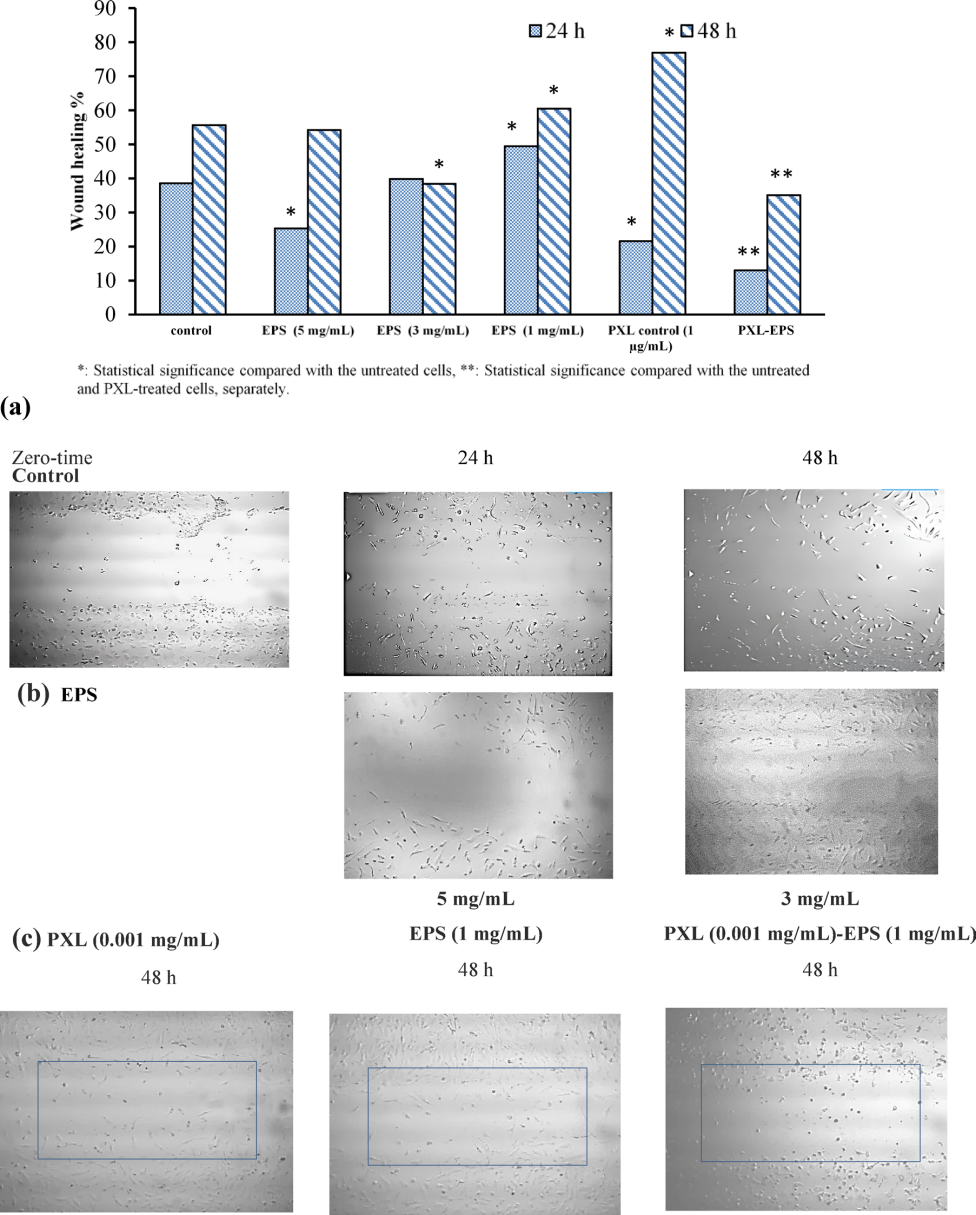
Antimigratory effects of PXL, EPS, and PXL–EPS combination treatments on MDA-MB-231 cells. (**a**) The statistical significance of each treatment as was determined using Student’s t test. (**b**) The antimigratory effects of 5 mg/mL EPS after 24 h and 3 mg/mL EPS after 48 h. (**c**) Antimigratory effects of PXL, EPS, and PXL**–**EPS after 48 h.

Interestingly, wounds treated with 1 mg/mL EPS showed a significantly reduced healing percentage according to the T score values calculated through Student’s t test after both 24 and 48 h (Fig. 13a). A relatively high concentration of 5 mg/mL EPS induced a significant reduction in the healing percentage only after 24 h, whereas 3 mg/mL EPS elicited a significant decline in the healing percentage after 48 h (Fig. 13a & b). The more pronounced antimigratory effect of the lowest EPS concentration (1 mg/mL) may be explained by a biphasic dose‒response of the EPS, as reported for some bioactive compounds. However, for most compounds showing biphasic responses, the lower concentrations are stimulating rather than inhibitory; thus, called hermetic concentrations^102^, while exceptionally for some biphasic responses, the lower concentration can be inhibitory, as in the current study and the study by Dang et al. (2003)^103^, where 0.1–1 µM genistein reduced adipocyte growth by 85%, whereas > 10 µM enhanced a 3.4-fold increase in adipogenesis in mesenchymal progenitors.

Notably, the combined treatment of PXL (0.001 mg/mL) and EPS (1 mg/mL) was associated with the most pronounced antimigratory potential with a statistically significant reduction in the migratory potential of the MDA-MB-231 cells compared with both the control cells and the PXL-treated (0.001 mg/mL) cells after 24 and 48 h (Fig. 13a & c). In addition, as shown by the morphology of the migratory cells inside the wounds, highlighted by the rectangular frames in Fig. 13c, the migratory cells in the PXL-treated wells showed a relatively healthier morphology, compared with the migratory cells in the PXL–EPS-treated wells, which appeared rounded, shrunk, darkened, and stressed, implying the enhancing action of low concentrations of EPS on the antimigratory action of the standard drug PXL.

- **Oxidative stress**,** apoptosis**,** and metastasis biomarker determination**.

It was intended to assess the levels of MDA and MMP-9 as well as casp-3 activity in the pellets or culture supernatants of MDA-MB-231 cells treated separately for 48 h with EPS (9.4 mg/mL), PXL (0.06 mg/mL), and a combination of PXL (0.03 mg/mL) and EPS (3 mg/mL). The individual and combined treatments were applied at their respective IC_50_ values, as determined by the Chou–Talalay median effect method.

**Fig. 14:**
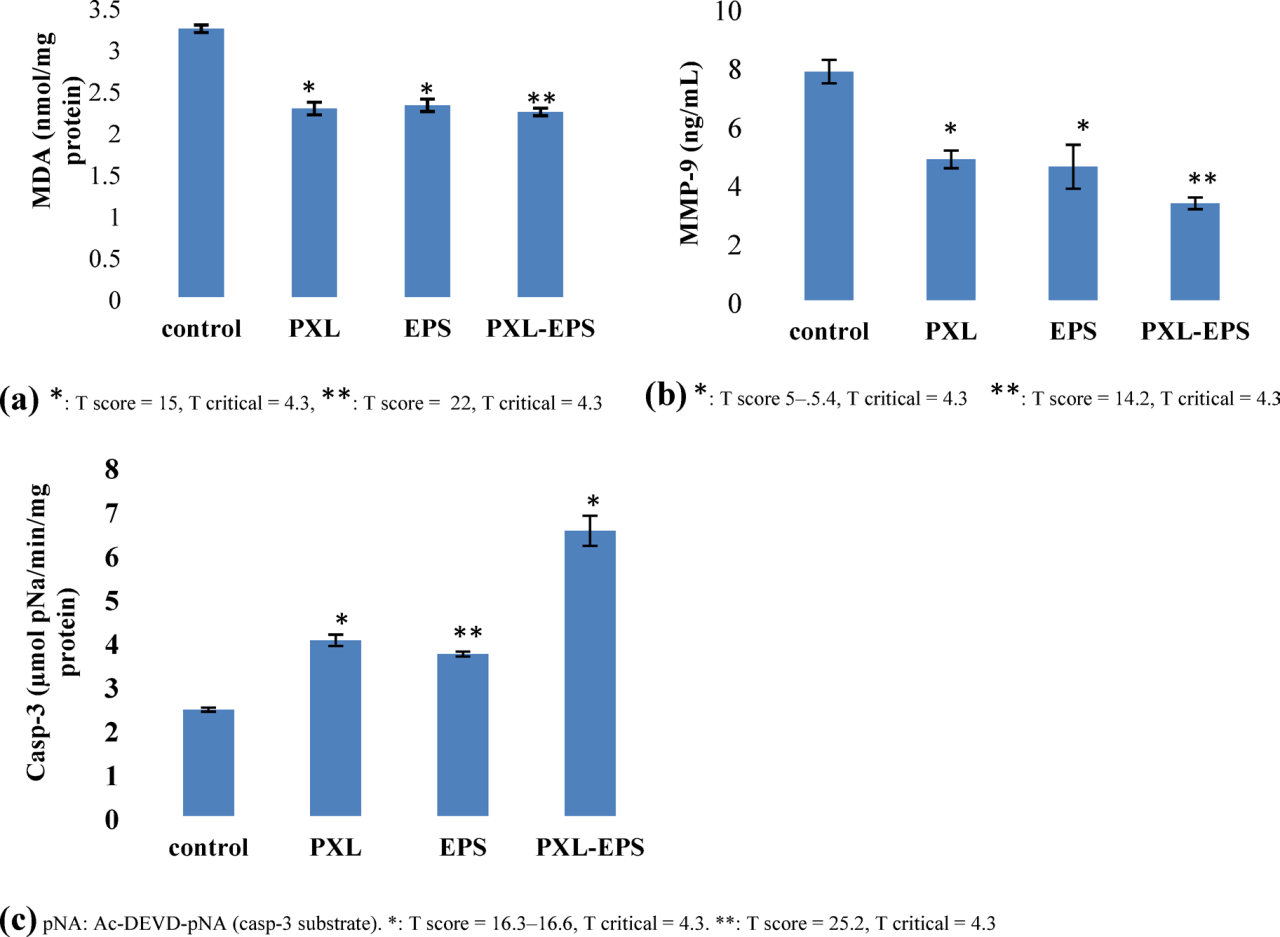
Effect of individual and EPS–PXL treatments on the MDA, MMP-9, and casp-3 levels in MDA-MB-231 cells. The error bars refer to the average deviation of the duplicate measurements.

Interestingly, the T scores calculated through the unpaired Student’s t test (α = 0.05) revealed that both EPS and the standard drug PXL significantly reduced the levels of the oxidative stress marker MDA in the MDA-MB-231 cells compared with those in the control cells, with PXL–EPS combined treatment showing the most significant reduction (Fig. 14a). Similarly, all the groups showed significant reductions in the levels of the metastasis marker MMP-9; nevertheless, PXL–EPS combined treatment exhibited the most significant reduction (Fig. 14b). The significant reduction in MPP-9 levels by EPS and PXL–EPS combination treatments aligns with the recorded antimigratory potential of both treatments, raising expectations of the EPS role in controlling TNBC metastasis. Interestingly, for the apoptosis marker casp-3, the EPS-treated cells displayed the most significant rise in casp-3 activity, followed by PXL–EPS and PXL-treated cells, which demonstrated a comparable statistical significance (Fig. 14c). The pro-apoptotic effect of EPS is consistent with its ability to induce cell cycle arrest at the S phase of growth.

In summary, the EPS of *N. chahannaoensis* BG8 displayed a pro-apoptotic action surpassing that of the standard drug PXL; furthermore, PXL–EPS combined treatment showed promise for reducing PXL-associated chemotherapy toxicity while better controlling TNBC metastasis, compared with PXL alone.

## Conclusion

Considering the limited research on haloarchaeal EPSs, the present study reports the isolation of a haloalkaliphilic archaeal strain, *N. chahannaoensis* BG8, which was not previously isolated from solar salterns on the northwestern Mediterranean coast of Egypt, considered an insufficiently explored extreme habitat in the country. This study revealed the production of EPS by *N. chahannaoensis* BG8 with anticancer attributes.

Notably, and to our knowledge, the present work records for the first time the anticancer activity of haloarchaeal EPS against the human breast cancer cell line MCF-7, the TNBC cells MDA-MB-231, the human squamous epidermoid carcinoma A-431 cells, and the human colorectal carcinoma HCT-116 cells, respectively. The anticancer activity was further supported by the recorded antimigratory action of the EPS against MDA-MB-231 cells, as demonstrated via the wound healing assay, and an S-phase cell cycle arrest, recorded through the flow cytometric analysis. Furthermore, the EPS interacted synergistically with the standard anticancer drug PXL in the MDA-MB-231 cells, an observation corroborated by the statistically significant pro-apoptotic, antimetastatic, and antimigratory effects of both the EPS and PXL‒EPS combined treatment, as shown by the recorded levels of casp-3 and MMP-9 and the results of the wound healing assay. In conclusion, the EPS of *N. chahannaoensis* BG8 displayed a pro-apoptotic effect surpassing that of the standard drug PXL; furthermore, PXL‒EPS combined treatment promises to reduce PXL-associated toxicity and better control TNBC metastasis; thus, raising expectations for the safe application of EPS as a complementary therapy more efficient than PXL alone.

Given the findings of the present work, more efforts should be made to mine the haloarchaeome of the less explored extreme saline habitats in Egypt and reveal the mechanistic and molecular basis of the anticancer action of haloarchaeal EPSs through both molecular docking and wet-laboratory investigations to better understand their health-protective application as an antioxidant and complementary therapy to improve the performance of chemotherapy and even mitigate its side effects. A special focus should be given to revealing the effects of PXL‒EPS combined therapy on the development of resistance towards PXL in cancer cells.

## Data Availability

Sequence data of the 16S rRNA gene of the study strain, which support the findings of this study, have been deposited into the GenBank database^104^ of the National Center for Biotechnology Information (NCBI) under accession number OR738703. Data are available under the following URL: https://sky-blast.com/blast/n/5e932a632dc0.
